# A systematic review and meta-analysis of the incidence rate of Takayasu arteritis

**DOI:** 10.1093/rheumatology/keab406

**Published:** 2021-05-04

**Authors:** Megan Rutter, Jonathan Bowley, Peter C Lanyon, Matthew J Grainge, Fiona A Pearce

**Affiliations:** 1 Department of Epidemiology and Public Health, University of Nottingham; 2 Department of Rheumatology, Nottingham University Hospitals NHS Trust, Nottingham, UK

**Keywords:** Takayasu arteritis, vasculitis, epidemiology, meta-analysis

## Abstract

**Objectives:**

Takayasu arteritis (TAK) is a rare autoimmune rheumatic disease causing large-vessel vasculitis. Onset is typically between the ages of 20 and 30 years. It is associated with substantial morbidity and mortality, notably due to its effects on the cardiovascular system. It has a poorly understood global epidemiology. Our objective was to systematically review the available evidence in order to calculate the incidence rate of TAK.

**Methods:**

Three databases (MEDLINE, PubMed and Embase) were searched in November 2019 and the results were screened by two reviewers. A random effects meta-analysis was then conducted in R to calculate the overall incidence rate. Heterogeneity was assessed using I^2^. The quality of the studies was assessed using an adapted Newcastle–Ottawa scale. Further subgroup analyses were performed by quality, sex, research setting and geographical location. Publication bias was assessed using a Begg’s funnel plot.

**Results:**

The incidence rate for TAK was 1.11 per million person-years (95% CI 0.70–1.76). The heterogeneity in the data was extremely high in all analyses, which suggests that there was considerable variation in incidence rates across the different populations studied. TAK was found to be more common in women (incidence rate 2.01 per million person-years, 95% CI 1.39–2.90).

**Conclusions:**

TAK is an extremely rare disease. It affects women more commonly than men. There is considerable variation in the incidence rate between populations. We suggest that future research should focus on discrete populations in order to better identify genetic and environmental risk factors.

**Rheumatology key messages**

- Takayasu arteritis is an ultra-rare disease, with implications for diagnosis, service design, treatment and research.
- Takayasu arteritis is more common in women than men and incidence differs across different populations.
- Accurate incidence data are important when designing and commissioning clinical services and treatments.

## Introduction

Takayasu arteritis (TAK) is a rare large-vessel vasculitis, which predominantly affects the aorta and its main branches. The disease and its sequelae lead to significant morbidity and mortality and may necessitate long-term immunosuppressive treatment [1, 2]. The global epidemiology of TAK remains unknown and there are no published systematic reviews with meta-analysis on the global incidence or prevalence of TAK. Prevalence seems to vary geographically and appears highest in Asia [3, 4].

The first-line treatment for TAK is CS, with the addition of a DMARD if there is a poor response to treatment [5]. Tocilizumab, an mAb against the IL-6 receptor, is now in use as a third-line therapy in some countries including England [5]. Many other drug pathways are under investigation, including TNF inhibition and Janus kinase inhibition.

The lack of clarity on the basic epidemiology of TAK has many implications, including on the design of diagnostic pathways, creation of specialized services, funding of high-cost drugs and on clinical trial recruitment. We aimed to perform a systematic review and meta-analysis of the incidence of TAK worldwide.

## Methods

The method for this systematic review was guided by the Preferred Reporting Items for Systematic reviews and Meta-Analyses (PRISMA) statement for systematic reviews [6]. The study protocol was registered on PROSPERO (an international prospective register of systematic reviews) on 9 December 2019 with registration number CRD42019138795.

The Population, Intervention, Comparison, Outcomes and Study (PICOS) framework was used to formulate the study protocol as follows. Population: all persons diagnosed with TAK. Papers that explored juvenile cases only were excluded. Outcome: incidence rate for TAK was the primary outcome measure. This review did not have an intervention or comparator group.

### Study eligibility

To meet the inclusion criteria, studies had to be cohort studies, report the incidence rate of TAK, and be published in English. Studies published prior to 1990, conference abstracts, reviews and studies exclusively focusing on disease in children aged <18 years were excluded.

### Data sources

The academic databases used for the systematic review were MEDLINE [MEDLINE 1946 to present (OVID)], Embase (OVID) and PubMed. Key words relating to the population and outcome of interest were developed using synonyms for each disease and their respective MeSH. A pilot search was performed independently by J.B. and F.A.P. in MEDLINE, to ensure specificity of the search. A list of seminal papers was used to test the sensitivity of the search and all were found. Subsequent searches with key words were independently performed by J.B. and F.A.P., with search terms developed for MEDLINE and further adapted for the other databases. Searches were carried out in November 2019. The search terms for MEDLINE and PubMed were (Takayasu.mp.) AND (*incidence/OR incidence.mp.) and for Embase (Takayasu.mp. OR aortic arch syndrome/) AND (incidence.mp. OR *incidence/) (supplementary Appendix S1, available at *Rheumatology* online). Records retrieved through our search were imported into the EndNote software, where duplicates were removed electronically, then manually.

### Study selection

Outputs from the literature search were screened in a three-stage logical manner, namely: title and abstract screening, full-text screening and extraction. Two reviewers (J.B. and F.A.P.) independently screened the retrieved studies. Conflicts in the study selection process were resolved by dialogue and Covidence was used to record decisions to include or exclude studies at each stage.

In one case where the full text was not immediately available, the text was obtained following correspondence with the author. Additional data beyond what was contained in the published manuscripts were not sought.

### Data extraction

Data extraction was conducted in parallel by two reviewers (J.B. and F.A.P.). Data regarding study details (author, year of publication), study characteristics (design, setting) and participant data (population size, male:female ratio, mean age at diagnosis, incidence rate for diseases studied and the corresponding 95% CIs) were extracted. The data extraction form used is shown in supplementary Appendix S2, available at *Rheumatology* online). Discrepancies were settled via dialogue.

The reviewers assessed the quality of selected studies using the Newcastle–Ottawa scale [7], adapted to fit this review (supplementary Appendix S3, available at *Rheumatology* online) Studies were assessed on the representativeness of the cohort, ascertainment of TAK diagnosis, demonstration that TAK diagnosis did not pre-date the start of the study, study population size, and whether the study design controlled for gender, ethnicity or age. The maximum number of points available was 5. Papers were grouped by number of points achieved, rather than in to high- or low-quality categories.

### Risk of bias assessment

Assessment of heterogeneity was performed using the I^2^ statistic. I^2^ >50% was taken to represent moderate heterogeneity, and I^2^ >75% to represent high heterogeneity. Publication bias within studies was assessed with a funnel plot using a random effects model.

### Data synthesis

The incidence rates were expressed per million person-years. Where papers did not provide the 95% CIs, they were calculated using the formulae below, where *D* is the number of cases of the disease.

$$\mathrm{Lower}\mathrm{confidence}\mathrm{interval}=\frac{\text{Incidence}\text{rate}}{\;\text{exp}\;(1.96\sqrt{(\frac{1}{D}}))}$$

$$\text{Upper}c\text{onfidence}\text{interval}=\text{incidence}\text{rate}\times \text{exp}\;(1.96\sqrt{(\frac{1}{D}}))$$

The distribution of incidence rates and 95% CIs obtained from the studies were examined using a forest plot. The log of the incidence rate and standard error were calculated for each study to allow these to be pooled using the inverse variance method. A random effects meta-analysis was used to pool incidence rates with application of the Hartung-Knapp-Sidik-Jonkman [8] method to adjust the CI of the pooled estimate.

Subgroup analysis was employed to explore the effect of geographical region, sex and the quality of the paper on incidence.

Many papers that reported the sex of the patients did not report the overall proportion of men and women in their populations. Where this was the case, a ratio of 50:50 was used as the estimated male:female ratio in the population. When calculating the effect of sex on the incidence, we found that several studies reported no cases among men. In this circumstance, in order to make meta-analysis possible, it is usual to put a rate slightly above zero in the group with no cases [9]. Therefore, where the incidence rate in the male subgroup was zero, an incidence rate of 0.5 per million person-years was used in order to allow meta-analysis. We must also consider in the case of a rare outcome whether arbitrarily adding to the numerator could, under random effects, cause an upward bias to the pooled estimate if we add 0.5 (or alternative quantity) to the zero event number for studies where a small denominator would mean that experiencing one or more events is unlikely. We took the pooled estimate for the annual incidence of 0.28 per million in males and applied this to the male denominators for the studies by Romero-Gomez *et al*. [10] and Nesher *et al*. [11] to estimate the expected number of events and then applied the Poisson formula. From this, the probability of obtaining one or more events in males if the underlying incidence in the Romero-Gomez *et al*. study [10] is the same as the pooled incidence is 0.47; therefore, adding 0.5 would not seem unreasonable. The equivalent probability for the study by Nesher *et al*. [11] is 0.51.

In light of the extreme heterogeneity observed in the initial analyses, a further subgroup analysis was performed by study setting (medical centre-based *vs* population-based). All statistical analyses were performed in R version 4.0.2 (packages *tidyverse*, *meta*, *metafor*).

## Results

The process for study selection is shown in a PRISMA flow diagram in supplementary Fig. S1, available at *Rheumatology* online. The initial database search returned 735 results. After duplicates were removed 519 papers remained, which were then screened. At the title and abstract stage, 505 papers were removed, leaving 14 for the full-text screening. A list of all excluded studies, including the reasons for exclusion, are available from the author on request.

Three papers were excluded at the full-text screening stage. One paper was a review and was therefore the wrong study design. One did not report the incidence rate for TAK, nor the background information that would have allowed us to calculate it. Finally, one was excluded as it investigated the incidence of TAK in children only.

Eleven studies met the inclusion criteria for the meta-analysis. Supplementary Table S1, available at *Rheumatology* online, provides an overview of their characteristics.

Five of the studies derived their data from population-based databases, two were single centre-based studies and the remaining four included multiple centres. The countries of origin were Turkey, Denmark, Norway, Poland, Australia, Sweden, Israel, South Korea, Spain and the UK. It is notable that most of the data came from Europe (especially Scandinavia) and that only one study (Park *et al.* [12]) looked at the incidence of TAK in Asia, where it was originally described.

Seven of the studies identified patients using local or national databases containing International Classification of Diseases (ICD)-8, -9 or -10 diagnostic codes, depending on the time period of the study. Makin *et al.* [13] used a mixture of ICD-10 diagnostic codes, key word searches in records of outpatient communication and direct contact with relevant local specialists to identify patients. One study (Watts *et al.* [14]) used the read code for TAK used in the national UK General Practice Research Database. One study (Park *et al.* [12]) identified patients from a national database in Korea called the Rare Intractable Disease (RID) registration programme. Nesher *et al.* [11] identified patients from local clinical databases.

Confirmation of TAK diagnosis was performed by 10 of the 11 studies, using a variety of methods. Seven used the 1990 ACR diagnostic criteria, one the modified Ishikawa criteria and one a combination of the two. One study included patients fulfilling either the 1990 ACR diagnostic criteria or the Chapel Hill Consensus Criteria for TAK. Park *et al.* [12] used the RID diagnostic criteria for TAK, which are similar to the ICD-10 diagnostic criteria other than for age. Kanecki *et al.* [15] accessed anonymized patient data and did not confirm the diagnosis from patient records.

**Fig. 1:**
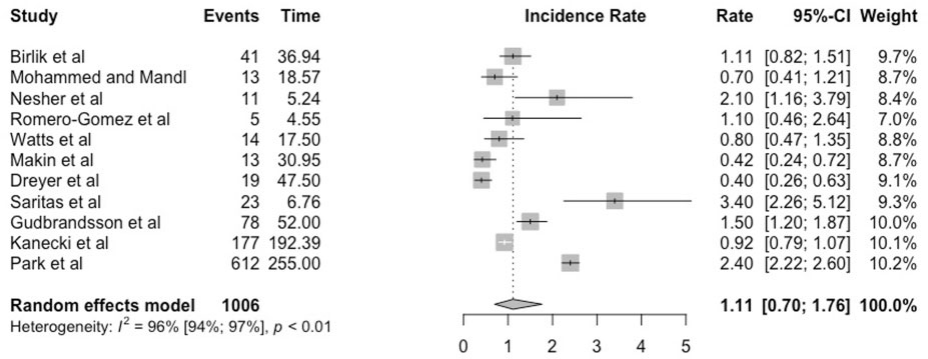
Forest plot showing incidence rate of Takayasu’s arteritis expressed per million person-years, with 95% CI

The meta-analysis of the incidence rate for TAK is shown in the forest plot in Fig. 1. The random effects meta-analysis estimated that the pooled-incidence rate of TAK was 1.11 per million person-years (95% CI 0.70–1.76).

There was high heterogeneity among the studies pooled for the meta-analysis (I^2^ = 96%). The I^2^ statistic describes the percentage of variation across studies that is due to heterogeneity rather than chance.

**Fig. 2:**
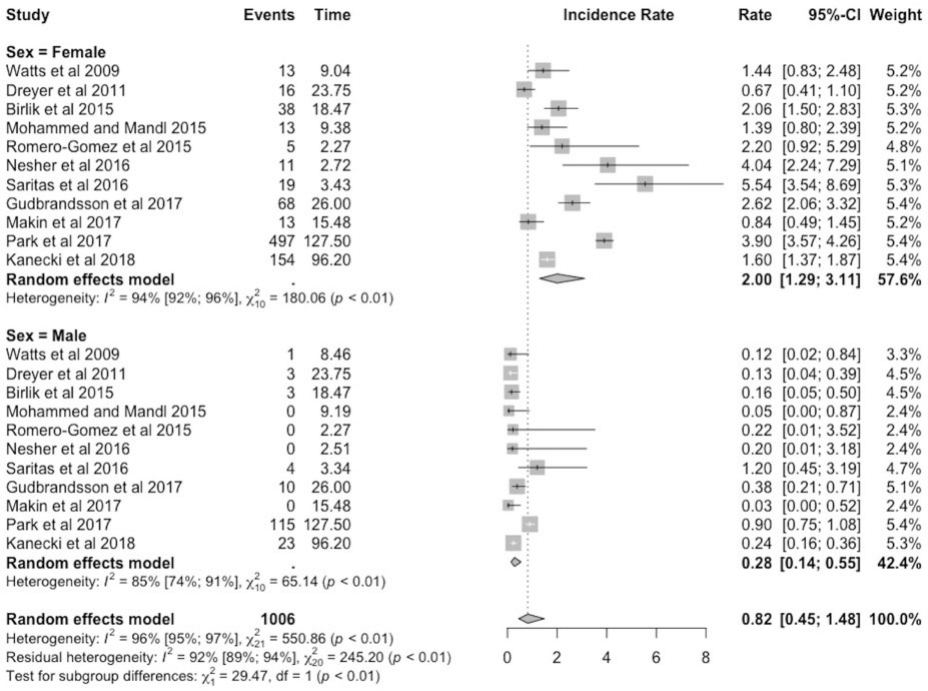
Forest plot of the subgroup analysis by sex, with incidence of Takayasu’s arteritis expressed per million person-years, with 95% CI

A subgroup analysis was then performed by sex. The results are shown in a forest plot in Fig. 2. The pooled incidence rate was higher for women (2.00 per million person-years, 95% CI 1.29–3.11) than for men (0.28 per million person-years, 95% CI 0.14–0.55). The heterogeneity also remained high, with I^2^ values of 94% (women) and 85% (men).

**Fig. 3:**
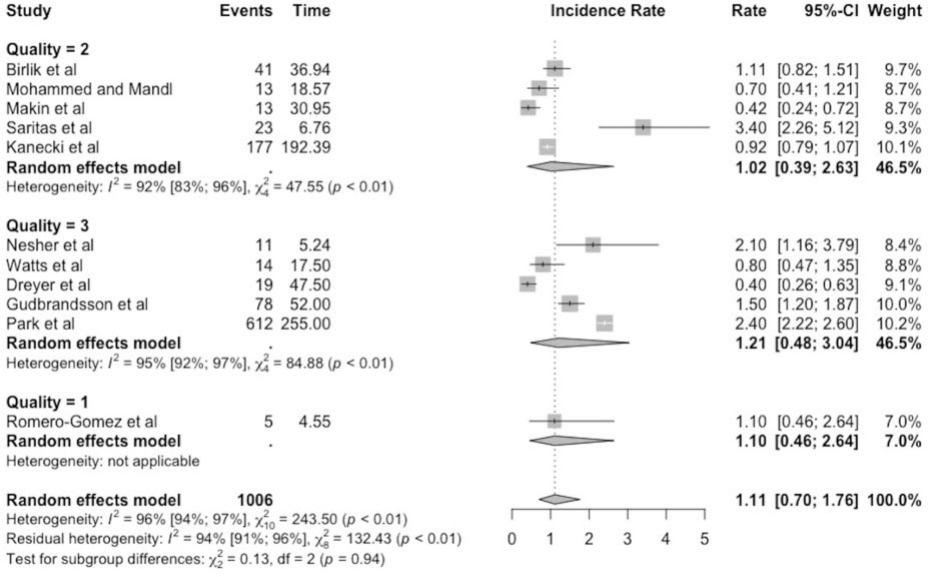
Subgroup analyses based on quality, with incidence of Takayasu’s arteritis expressed per million person-years, with 95% CI

The results grouped by quality assessment are presented in a forest plot in Fig. 3. Out of a total of 5 possible points for quality, no papers scored 5 or 4, five papers scored 3, five papers scored 2 and one paper scored 1. The incidence rates with 95% CIs were 1.02 (0.39–2.63) and 1.21 (0.48–3.04), respectively, for papers that scored 2 and 3. Given that the 95% CIs overlapped, any difference in incidence rate may be due to chance. Heterogeneity remained high with I^2^ values of 92% and 95% in the 2- and 3-point groups, respectively.

**Fig. 4:**
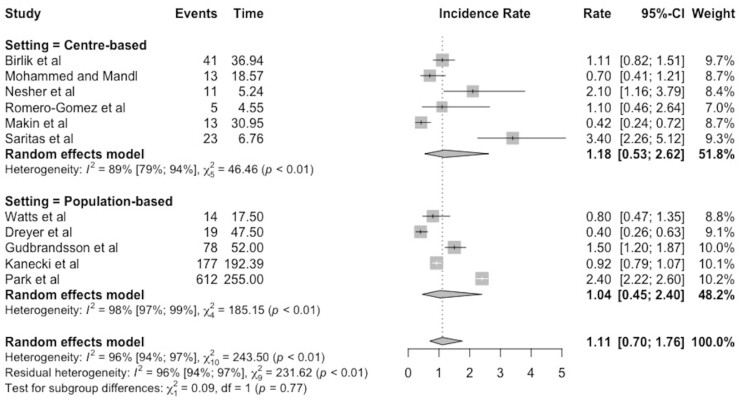
Forest plot of the subgroup analysis based on setting, with incidence of Takayasu’s arteritis expressed per million person-years, with 95% CI

The studies included in this meta-analysis were classified as medical centre-based or population-based settings, and separate subgroup analyses based on these categories are shown in the forest plot in Fig. 4. For medical centre-based studies, the incidence rate and corresponding 95% CIs were 1.18 (0.53–2.62) and for population-based studies 1.04 (0.45–2.40). The CIs overlap, so the difference in the incidence rates may be due to chance. The high heterogeneity observed remained in both subgroups, with I^2^ values of 89% for centre-based studies and 98% for population-based studies.

**Fig. 5:**
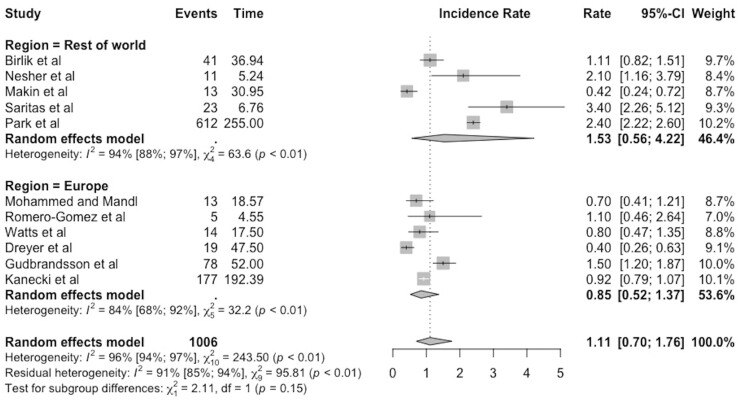
Subgroup analyses of studies based on geographical location, with incidence of Takayasu’s arteritis expressed per million person-years, with 95% CI

The studies were also analysed according to geographical region and the results are shown in Fig. 5. The incidence rate and corresponding 95% CIs were 0.85 (0.52–1.37) for European studies and 1.53 (0.56–4.22) for studies from the rest of the world. The CIs overlap, so the difference in the incidence rates may be due to chance. The high heterogeneity observed remained in both subgroups, with I^2^ values of 84% for Europe and 94% for the rest of the world.

An influence analysis was performed to assess the effect of each study on the overall pooled incidence rate. The systematic exclusion of each study lead to no clinically meaningful effect on the pooled incidence rate, with the results from the 11 separate analyses with a different study omitted each time ranging from 1.00 (0.64–1.54) to 1.23 (0.79–1.94).

**Fig. 6:**
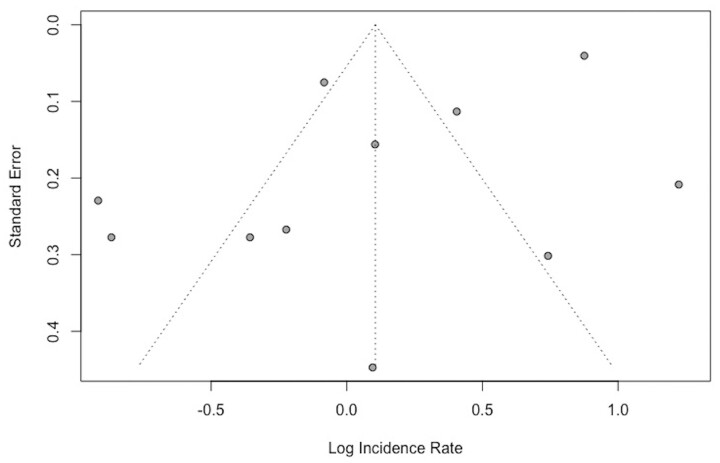
Begg’s funnel plot of the studies investigating the incidence of Takayasu’s arteritis included in the meta-analysis

Publication bias was assessed using a Begg’s funnel plot, which is shown in Fig. 6. The results were distributed symmetrically, suggesting a lack of publication bias. Seven of the studies fell outside the pseudo 95% CIs.

## Discussion

### Main findings

This study is, to the best of our knowledge, the first to examine the global incidence of TAK. The meta-analysis conducted shows TAK to be an exceedingly rare disease with an incidence rate of 1.11 (95% CI 0.70–1.76) per million person-years. Our point estimates suggest that TAK is six times more common in women than men.

### Heterogeneity in the literature

Whilst TAK is exceedingly rare in all studied populations, there was high heterogeneity in incidence rates between studies. We considered whether this was due to methodology by conducting subgroup analyses by research setting (medical centre- or population-based) and research quality. These analyses did not reduce the heterogeneity seen. The heterogeneity was partially explained by sex but remained high in both the male and female subgroups. This suggests a true difference in the incidence of TAK between different populations. Data were not available to analyse by race.

### Strengths and potential limitations

To our knowledge, this is the first systematic review of studies of the incidence of TAK.

Studies were restricted to those published in English where no suitable translation was available. Whilst this is fairly common practice with systematic reviews, particularly due to the increasing shift to publishing in English internationally, it is possible that relevant studies could have been missed. No grey literature was searched, which could have provided additional studies for screening and potential inclusion.

This review was limited by the quality of the studies included in the meta-analysis. In our adapted Newcastle–Ottawa scale, the maximum score was 5, which no studies achieved. Most studies scored either 2 or 3, indicating average or poor quality.

The extreme heterogeneity shown in all forest plots was not limited by sex, study quality, setting or geographical region. This demonstrates the limited utility of attempting to calculate global incidence rates for diseases, which are likely to vary in their incidence in different populations. We suggest that future epidemiological studies focus on discrete populations and carry out thorough subgroup analyses to further assess the factors that lead to variation.

Finally, our funnel plot analysis may have been insufficient to rule out the potential for publication bias. Whilst presence of asymmetry in a funnel plot can be used to identify possible publication bias along with other problems, these can be unreliable when the number of studies is small (*n* = 11 in this case). We believe our findings may have been affected by publication bias as studies that provide data showing TAK is more common than previously thought may be more likely to be published than those that report it to be more rare. Furthermore, studies may be more likely to be conducted and reported in populations where TAK is more common, which has implications if we then generalize this to the incidence of TAK worldwide.

### Further research

There is a need for research that determines the incidence of TAK in different geographical areas. At the moment the research is dominated by data from Northern Europe and Turkey. We did not identify any studies from South America or Africa. Sub-analysis of geographical area showed a trend towards lower incidence rates in Europe when compared with the rest of the world. However, the overlap in CIs means that this could have occurred through chance.

### Clinical implications

Tocilizumab has recently been commissioned by NHS England as a third-line treatment for TAK in adults ‘where attempts to control disease progression of TAK have failed’ [5]. It is estimated that up to 50% of patients with TAK may benefit from a biologic agent [5]. The commissioning policy is based on the incidence of TAK in Southern Sweden, from 13 patients identified between 1997 and 2011 in a population of almost 1 million [16]. In this way, TAK is an exemplar for many rare rheumatic diseases where we lack epidemiological data to guide healthcare planning for conditions that require highly specialized treatment and concentrated clinical knowledge and resources.

To provide more accurate incidence data for these rare diseases, we need contemporary whole population-based studies. This has recently been made possible by the creation of the National Congenital Anomaly and Rare Disease Registration Service by Public Health England and the Registration in Complex Rare Diseases—Exemplars in Rheumatology (RECORDER) project, which aims to identify and build a register of those individuals living with rare diseases, including rare autoimmune rheumatic diseases, amongst the 55 million people living in England [17, 18].

### Conclusions

This systematic review of the literature has demonstrated that the incidence rate of TAK is 1.11 (95% CI 0.70–1.76) cases per million person-years. TAK is more common in women than men. There appears to be a genuine difference in incidence in different populations. The reasons for this warrant further research.

*Funding statement*: M.R. is funded by Vasculitis UK (patient charity). F.A.P. and P.C.L. are recipients of a grant from Vifor pharma. Vifor pharma had no influence on the design, conduct or interpretation of this study.

*Disclosure statement*: F.A.P. and P.C.L. are recipients of a grant from Vifor pharma. Vifor pharma had no influence on the design, conduct or interpretation of this study.

## Data availability statement

The data underlying this article will be shared on reasonable request to the corresponding author.

## Supplementary data

Supplementary data are available at *Rheumatology* online.

## Supplementary Material

keab406_Supplementary_Data

## References

[1] Kerr GS , Hallahan CW, Giordano J et al Takayasu arteritis. Ann Intern Med 1994;120:919–29. 7909656 10.7326/0003-4819-120-11-199406010-00004

[2] Maksimowicz-McKinnon K , Clark TM, Hoffman GS. Limitations of therapy and a guarded prognosis in an American cohort of Takayasu arteritis patients. Arthritis Rheum 2007;56:1000–9. 17328078 10.1002/art.22404

[3] Onen F , Akkoc N. Epidemiology of Takayasu arteritis. Presse Med 2017;46:e197–203. 28756072 10.1016/j.lpm.2017.05.034

[4] Koide K. Takayasu arteritis in Japan. Heart Vessels Suppl 1992;7:48–54. 1360971 10.1007/BF01744544

[5] NHS England Clinical Commissioning Policy: Tocilizumab for Takayasu arteritis (adults). Reference: NHS England: 16056/P. 2016. https://www.england.nhs.uk/wp-content/uploads/2018/07/Tocilizumab-for-Takayasu-arteritis.pdf (14 June 2021, date last accessed)

[6] Moher D , Shamseer L, Clarke M et al Preferred reporting items for systematic review and meta-analysis protocols (PRISMA-P) 2015 statement. Syst Rev 2015;4:1–9. 25554246 10.1186/2046-4053-4-1 PMC4320440

[7] Wells GA , Shea B, O’Connell DA et al. The Newcastle-Ottawa Scale (NOS) for assessing the quality of nonrandomised studies in meta-analyses. Published 1 February 2000. http://www.ohri.ca/programs/clinical_epidemiology/oxford.htm (14 June 2021, date last accessed).

[8] IntHout J , Ioannidis JP, Borm GF. The Hartung-Knapp-Sidik-Jonkman method for random effects meta-analysis is straightforward and considerably outperforms the standard DerSimonian-Laird method. BMC Med Res Methodol 2014;14:25. Dec 24548571 10.1186/1471-2288-14-25 PMC4015721

[9] Lane PW. Meta-analysis of incidence of rare events. Stat Methods Med Res 2013;22:117–32. Apr 22218366 10.1177/0962280211432218

[10] Romero-Gomez C , Aguilar-Garcia JA, Garcia-de-Lucas MD et al Epidemiological study of primary systemic vasculitides among adults in southern Spain and review of the main epidemiological studies. Clin Exp Rheumatol 2015;33(2 Suppl 89):11–8. 25437862

[11] Nesher G , Ben-Chetrit E, Mazal B, Breuer GS. The incidence of primary systemic vasculitis in Jerusalem: a 20-year hospital-based retrospective study. J Rheumatol 2016;43:1072–7. 27084915 10.3899/jrheum.150557

[12] Park SJ , Kim HJ, Park H et al Incidence, prevalence, mortality and causes of death in Takayasu Arteritis in Korea - A nationwide, population-based study. Int J Cardiol 2017;235:100–4. 28283361 10.1016/j.ijcard.2017.02.086

[13] Makin K , Isbel M, Nossent J. Frequency, presentation, and outcome of Takayasu arteritis in Western Australia. Mod Rheumatol 2017;27:1019–23. 28350246 10.1080/14397595.2017.1300083

[14] Watts R , Al-Taiar A, Mooney J, Scott D, Macgregor A. The epidemiology of Takayasu arteritis in the UK. Rheumatology (Oxford) 2009;48:1008–11. 2009 19542212 10.1093/rheumatology/kep153

[15] Kanecki K , Nitsch-Osuch A, Tyszko PZ et al Takayasu’s arteritis: a rare disease in Poland. Ann Agric Environ Med 2018;25:469–72. 30260188 10.26444/aaem/92702

[16] Mohammad AJ , Mandl T. Takayasu arteritis in southern Sweden. J Rheumatol 2015;42:853–8. 25774057 10.3899/jrheum.140843

[17] Pearce FA , Rutter M, Griffiths B et al O36 Validation of methods to enable national registration for rare autoimmune rheumatic diseases. Rheumatology (Oxford) 2020;59: 10.1093/rheumatology/keaa110.035.

[18] Peach E , Rutter M, Lanyon P et al Risk of death among people with rare autoimmune diseases compared to the general population in England during the 2020 COVID-19 pandemic. Rheumatology (Oxford) 2021;60:1902–9. 33271595 10.1093/rheumatology/keaa855 PMC7798585

