# Supplementary material for: A systematic review and meta-analysis of the incidence rate of Takayasu arteritis
Source: Rheumatology (Oxford). 2021 May 4;60(11):4982–90. doi: 10.1093/rheumatology/keab406 (PMC8566298; doi:10.1093/rheumatology/keab406)
Supplement: keab406_Supplementary_Data [file keab406_supplementary_data.zip › keab406-suppl_data/rhe-20-3073-File009.docx]

**Appendix 1. Adapted Newcastle-Ottawa scale.**

**COHORT STUDIES**

Note: A study can be awarded a maximum of one star for each numbered item.

**Selection**

1) Representativeness of the cohort

a) truly representative ✵

b) somewhat representative ✵

d) no description of the derivation of the cohort

2) Ascertainment of exposure

a) secure record (e.g. surgical records) ✵

c) written self-report

d) no description

3) Demonstration that outcome of interest was not present at start of study

a) yes ✵

b) no

4) Was the study population large enough?

a) >1,000,000 ✵

b) <1,000,000

**Comparability**

1) Comparability of cohorts on the basis of the design or analysis

a) Study controls for gender, ethnicity or age (select the most important factor) ✵

**Appendix 2. Data extraction form**

| Author(s) | Year | Type of Study | Setting | Population | Age | incidence/1.000,000 | LCI | UCI |
| --- | --- | --- | --- | --- | --- | --- | --- | --- |
|  |  |  |  |  |  |  |  |  |
| Female:male | logir | loguci | loglci | Quality | Male ir | Male uci | Male lci | Male logir |
|  |  |  |  |  |  |  |  |  |
| Male loguci | Male loglci | Female ir | Female uci | Female lci | Female logir | Female loguci | Female loglci |  |
|  |  |  |  |  |  |  |  |  |

**Figure 1. PRISMA flow diagram for study screening and selection for Systematic Review and Meta-analysis of TAK.**


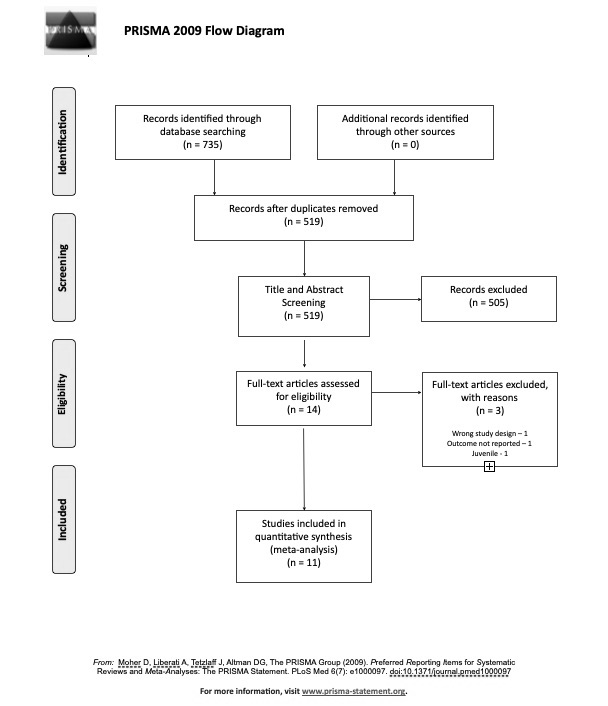


| **Author(s)** | **Year** | **Country** | **Study Design** | **Setting** | **Sample Size** | **Female: Male** | **Age at**  **Diagnosis**^†^ | **Quality Score** | **Diagnostic criteria** |
| --- | --- | --- | --- | --- | --- | --- | --- | --- | --- |
| Watts et al | 2009 | UK | Cohort | Population-based | 14 | 13:1 | 51 | 3 | UKGPRD |
| Dreyer, Farschou and Baslund | 2011 | Denmark | Cohort | Population-based | 19 | 16:3 | 36^†^ | 3 | Modified Ishikawa |
| Birlik et al | 2015 | Turkey | Cohort | Centre-based | 41 | 38:3 | 37.2 | 2 | ACR |
| Mohammed and Mandl | 2015 | Sweden | Cohort | Centre-based | 13 | 13:0 | 23^†^ | 3 | ACR |
| Romero-Gomez et al | 2015 | Spain | Retrospective Cohort | Centre-based | 5 | 5:0 | 26 | 1 | ACR |
| Nesher et al | 2016 | Israel | Retrospective Cohort | Centre-based | 11 | 11:0 | 34 | 2 | ACR or CHCC |
| Saritas et al | 2016 | Turkey | Cohort | Centre-based | 23 | 19:4 | Not reported | 2 | ACR |
| Gudbrandsson et al | 2017 | Norway | Cohort | Population-based | 78 | 68:10 | 33.9 | 3 | ACR or modified Ishikawa |
| Makin, Isbel and Nossent | 2017 | Australia | Retrospective Cohort | Centre-based | 13 | 13:0 | 39^†^ | 2 | ACR |
| Park et al | 2017 | South Korea | Cohort | Population-based | 612 | 497:115 | 46 | 3 | RID |
| Kanecki et al | 2018 | Poland | Cohort | Population-based | 177 | 154:23 | 45.4 | 2 | Not performed |

***Table 1. Summary of the characteristics of studies on incidence of TAK eligible for systematic review and meta-analysis.***

*Age:* † *denotes median, otherwise given as mean. UKGPRD = United Kingdom General Practice Research Database read code for TAK. Modified Ishikawa = Modified Ishikawa diagnostic criteria for TAK. ACR = 1990 American College of Rheumatology diagnostic criteria for TAK. CHCC = Chapel Hill Consensus Criteria for TAK. RID = Rare Intractable Disease registration programme criteria.*
