# Supplementary figures and images for: A systematic review and meta-analysis of the incidence rate of Takayasu arteritis
Source: Rheumatology (Oxford). 2021 May 4;60(11):4982–90. doi: 10.1093/rheumatology/keab406 (PMC8566298; doi:10.1093/rheumatology/keab406)

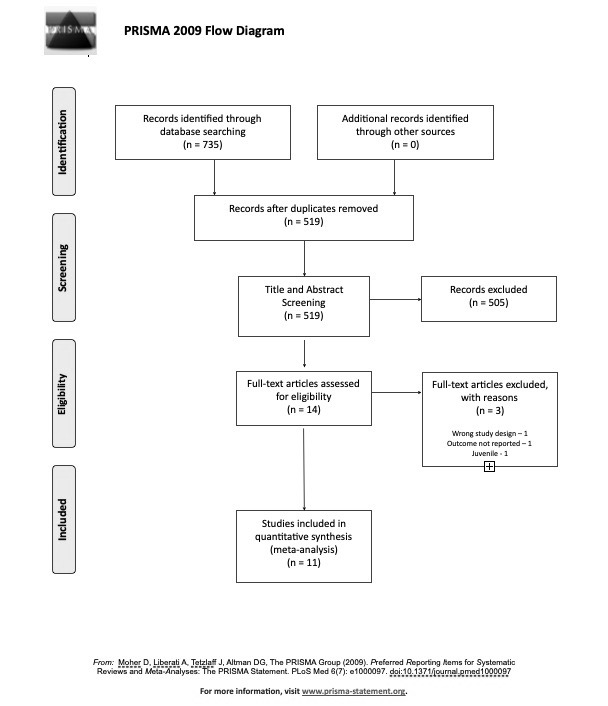

Supplement: keab406_Supplementary_Data [file keab406_supplementary_data.zip › keab406-suppl_data/rhe-20-3073-File002.jpeg]
